# Magma dynamics within a basaltic conduit revealed by textural and compositional features of erupted ash: the December 2015 Mt. Etna paroxysms

**DOI:** 10.1038/s41598-017-05065-x

**Published:** 2017-07-06

**Authors:** Massimo Pompilio, Antonella Bertagnini, Paola Del Carlo, Alessio Di Roberto

**Affiliations:** Istituto Nazionale di Geofisica e Vulcanologia, Sezione di Pisa, Italy

## Abstract

In December 2015, four violent explosive episodes from Mt. Etna’s oldest summit crater, the Voragine, produced eruptive columns extending up to 15 km a.s.l. and significant fallout of tephra up to a hundred km from the vent. A combined textural and compositional study was carried out on pyroclasts from three of the four tephra deposits sampled on the volcano at 6 to 14 km from the crater. Ash fractions (Φ = 1–2) were investigated because these grain sizes preserve the magma properties unmodified by post- emplacement processes. Results were used to identify processes occurring in the conduit during each single paroxysm and to understand how they evolve throughout the eruptive period. Results indicate that the magmatic column is strongly heterogeneous, mainly with respect to microlite, vescicle content and melt composition. During each episode, the heterogeneities can develop at time scales as short as a few tens of hours, and differences between distinct episodes indicate that the time scale for completely refilling the system and renewing magma is in the same order of magnitude. Our data also confirm that the number and shape of microlites, together with melt composition, have a strong control on rheological properties and fragmentation style.

## Introduction

In the last three decades Mt. Etna (Italy) has been characterized by almost continuous mild explosive activity punctuated by more violent paroxysms of sub-Plinian intensity^1, 2^. Volcanological and petrological studies of explosive products have traditionally been used to shed light on magma ascent and eruption dynamics. At Mt. Etna textural and compositional studies have mainly concentrated on explosive activity from fissures or cones located on the flanks of the volcano (e.g. 2001–2003)^3–5^. More energetic eruptions from the volcano’s summit have received relatively little attention^6^. Here, we will focus on the most recent paroxysmal activity of the Voragine, the oldest summit crater of Mt. Etna. In the last two decades this crater had only shown degassing interrupted by large explosive episodes, (e.g. 24 July 1998 and 4 September 1999)^7^ and by a few short-lived periods of Strombolian activity as in March 2013 and January 2015.

Starting in the second half of October, the Voragine produced an intra-crater Strombolian activity that in November progressively increased and culminated in four violent explosive episodes occurred (Table 1) on December 3^rd^, 4^th^ and 5^th^ 2015. These episodes, each lasting 50–60 minutes, generated eruptive columns extending between 15 km (episode #1) and 12.5 km (episodes #3 and #4) a.s.l.^8^ (Table 1) and significant fallout of tephra up to one hundred kilometers away from the vent. Morphological changes in the summit of the volcano were also observed. The mass erupted decreased from 9 (episode #1) to 1.2 10^9^kg (episode #4). The peak mass discharge rate (MDR) also varied from ~6 (episode #1) to ~0.9 10^6^ kg s^−1^ (episodes #3 and #4)^8^ (Table 1).

**Table 1:** Timing and eruptive parameters from^8^.

	Start (GMT)	End (GMT)	Mass (10^9^ kg)	Max Column Height (km a.s.l.)	MDR peak (10^6^ kg s^−1^)
Episode #1	03/12/15 02:30	03/12/15 03:30	9	15	≈6
Episode #2	04/12/15 09:10	04/12/15 10:10	2.4	13.4	≈2
Episode #3	04/12/15 20:30	04/12/15 21:20	1.2	12.5	≈0.9
Episode #4	05/12/15 14:50	05/12/15 15:40	1.2	12.5	≈0.9

The mass of the magma erupted during such events exceeds that produced by Strombolian explosions (10^4^ kg^9^) by several orders of magnitude, thereby providing insight into a significant portion of the shallow plumbing system and yielding invaluable information on the physical properties of the magmatic column and, indirectly, on the late stages of magma ascent.

We report here the results of a combined textural and compositional study of tephra deposits collected along the dispersion axes on the flanks of the volcano. In particular, ash fractions (1–2 Φ) were investigated because they are not modified by post-emplacement processes and represent a record of the magma residing within the conduit just before and during the eruption.

Sampling of three of the four paroxysms allowed the definition of magma dynamics throughout the eruptive period. In addition, textural and compositional changes within a single explosive episode were investigated for the first time for this kind of eruptions through detailed vertical sampling of the deposit (episode #1), yielding important insights into how intra-eruptive processes develop in the conduit.

## Results

### Deposits

**Figure 1:**
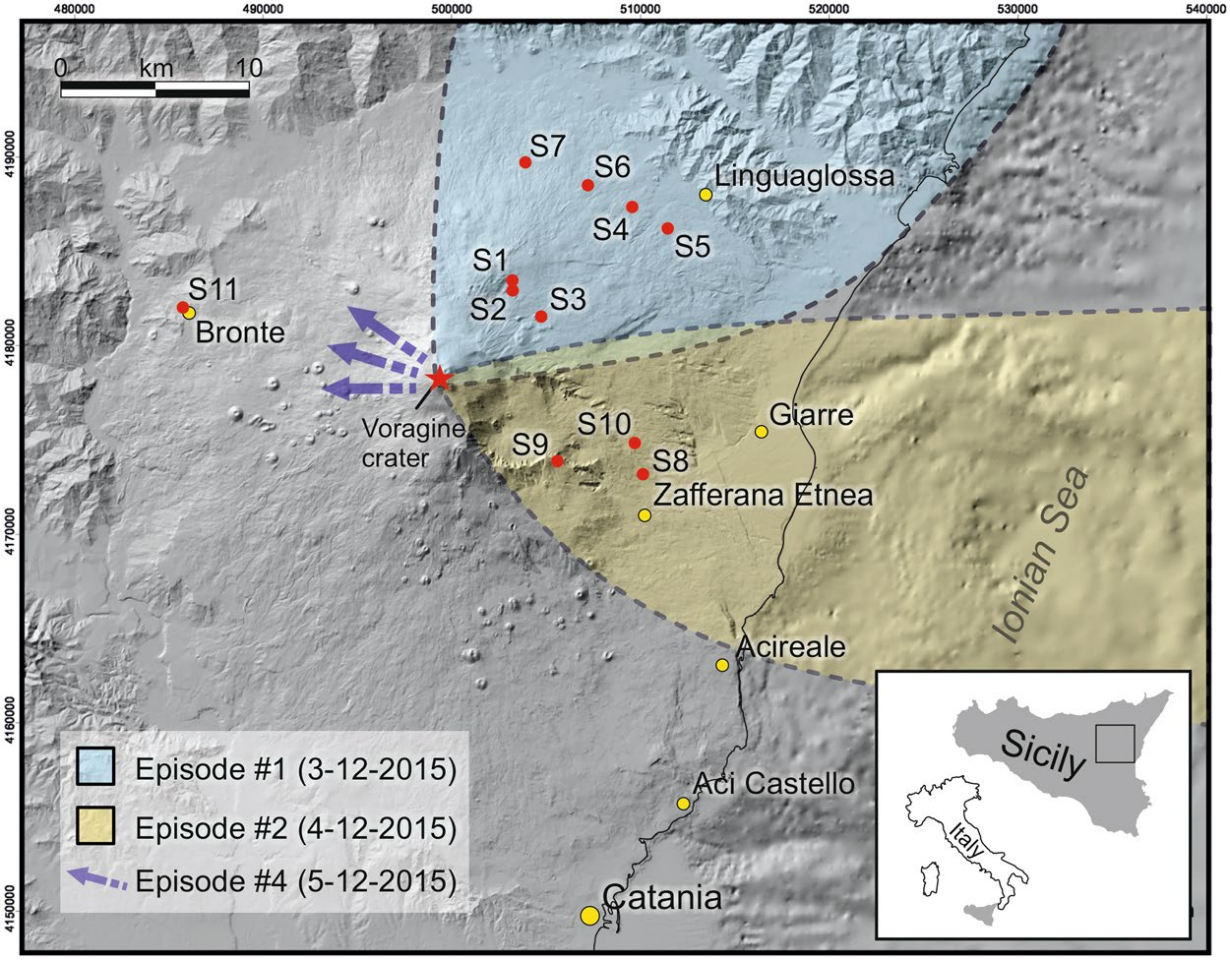
Map of Mt. Etna with sampling sites and dispersal areas of fallout deposits produced by explosive episodes occurring on 3–5 December 2015. Base map reprinted and modified from Journal of Volcanology and Geothermal Research Vol 251, Argnani, A., Mazzarini, F., Bonazzi, C., Bisson, M. & Isola, I. The deformation offshore of Mount Etna as imaged by multichannel seismic reflection profiles pp 50–64. Copyright (2013) with permission from Elsevier.

Fallout deposits from the four episodes had different dispersal. Products emitted during episode #1 dispersed toward the NE, reaching the city of Reggio Calabria (70 km from the volcano), whereas those from episode #2 fell on the eastern sector of the volcano. The direction of the eruptive plume from episode #3 was intermediate to that of the two preceding episodes, and that from episode #4 was to the west (D. Andronico, personal communication, INGV-OE reports) (Fig. 1).

Up to a distance of about 13 km from the vent, the tephra of episode #1 formed a nearly continuous cm/mm-thick blanket of black scoria. At sites 1 and 2 (Fig. 1) the deposit was 3.5 cm and 5 cm thick, respectively (Supplementary Figure 1), and consisted of lapilli and coarse ash. Grain size analysis revealed a reverse grading (Supplementary Table 1), as also indicated by the occurrence of scattered, cm-sized (up to 5 cm) scoria clasts at the top of the deposit (Supplementary Figure 1).

At comparable distances from the vent, the deposit of episode #2 was similar to that of episode #1, except for its finer fraction consisting of coarse ash.

As for episode #4, the deposit was identified only in the town of Bronte (Fig. 1, Supplementary Table 1), where it consists of a tiny layer of fine black ash. The juvenile component is dominant in all deposits and there are only very rare lithics, mainly consisting of reddened scoriae and lava fragments.

Due to the limited number of sampling sites, it was impossible to draw an isopach map and to calculate volumes.

### External morphology of pyroclasts

**Figure 2:**
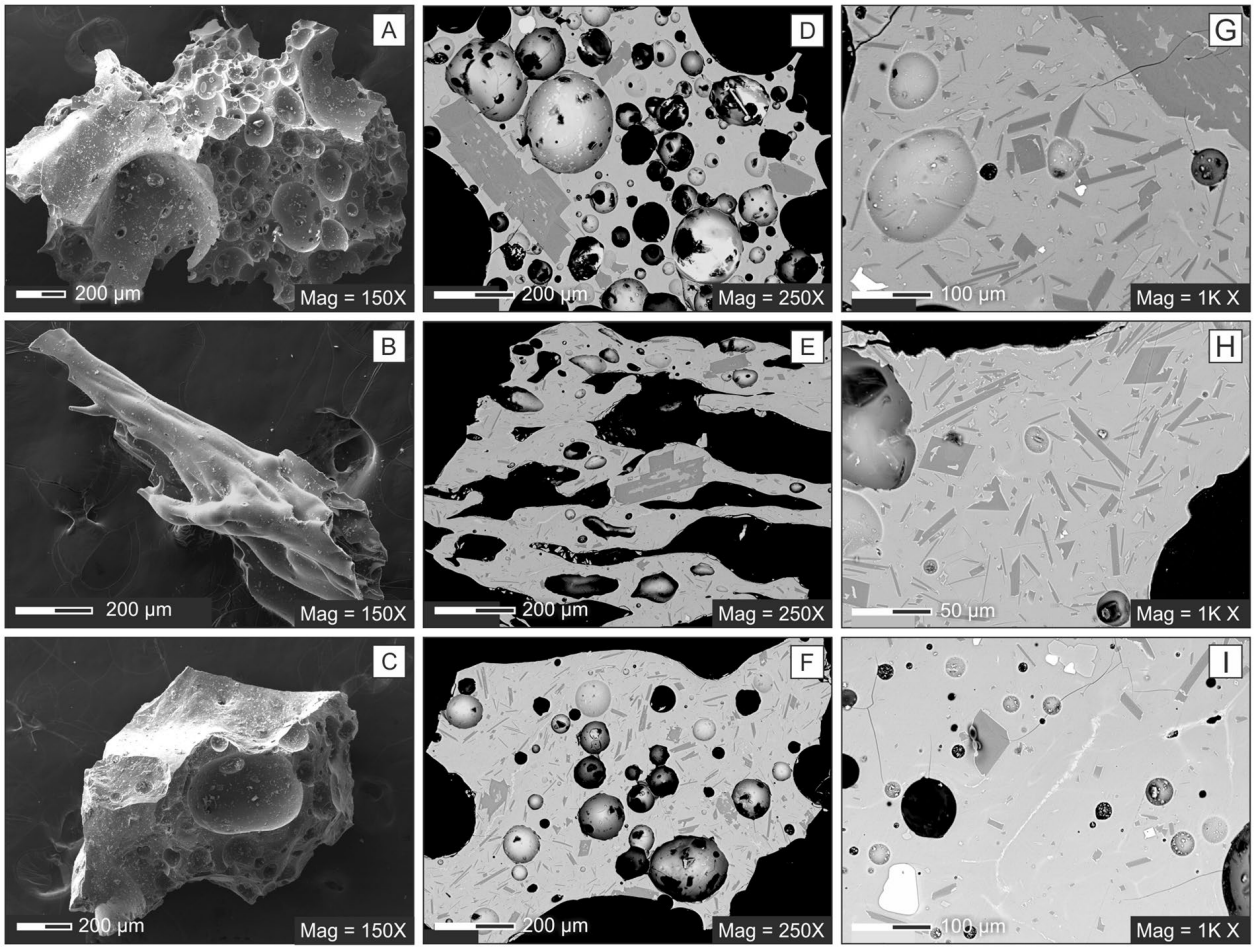
SEM images of ash fractions showing the external morphology of clasts (A = spongy, B = fluidal, C = blocky), vesicularity (D = moderate, E = poor, F = incipient), groundmass crystallinity (G = highly crystalline, H = moderately crystalline; I = poorly crystalline).

Three main classes of juvenile fragments were identified on the basis of their external morphology: a) spongy, b) fluidal, and c) blocky (Fig. 2 and Supplementary Figure 2). Spongy clasts are equant to sub-equant with rough, curved and irregular shapes mainly determined by the rupture of randomly oriented spherical to elliptical vesicles. Vesicularity varies from poor to moderate^10^. In the more vesicular particles, vesicles are often coalescent. Fluidal clasts have elongated, fluidal shapes with curved, smooth surfaces that do not intersect vesicles, as commonly observed in low-viscosity magma pyroclasts (achneliths^11^). External surfaces often exhibit wrinkles parallel or perpendicular to the particle elongation direction (Supplementary Figure 2G). For many particles, the elongated shape is often the result of interconnected tubular vesicles (Fig. 2B). Occasionally, small nearly-spherical vesicles grow in septa that separate tubes. Vesicularity varies from incipient to poor^10^. Cracks several tens of microns long often occur perpendicular to the particle elongation direction. Blocky clasts show generally equant to sub-equant shapes and have smoothed, curved or fracture-bounded surfaces (Fig. 2C and Supplementary Figure 2E and F). In these clasts, vesicularity ranges from incipient to moderate^10^ and bubbles are sub-spherical, elongated or amoeboid, and collapsed. Randomly oriented cracks several tens of microns in size often occur.

**Figure 3:**
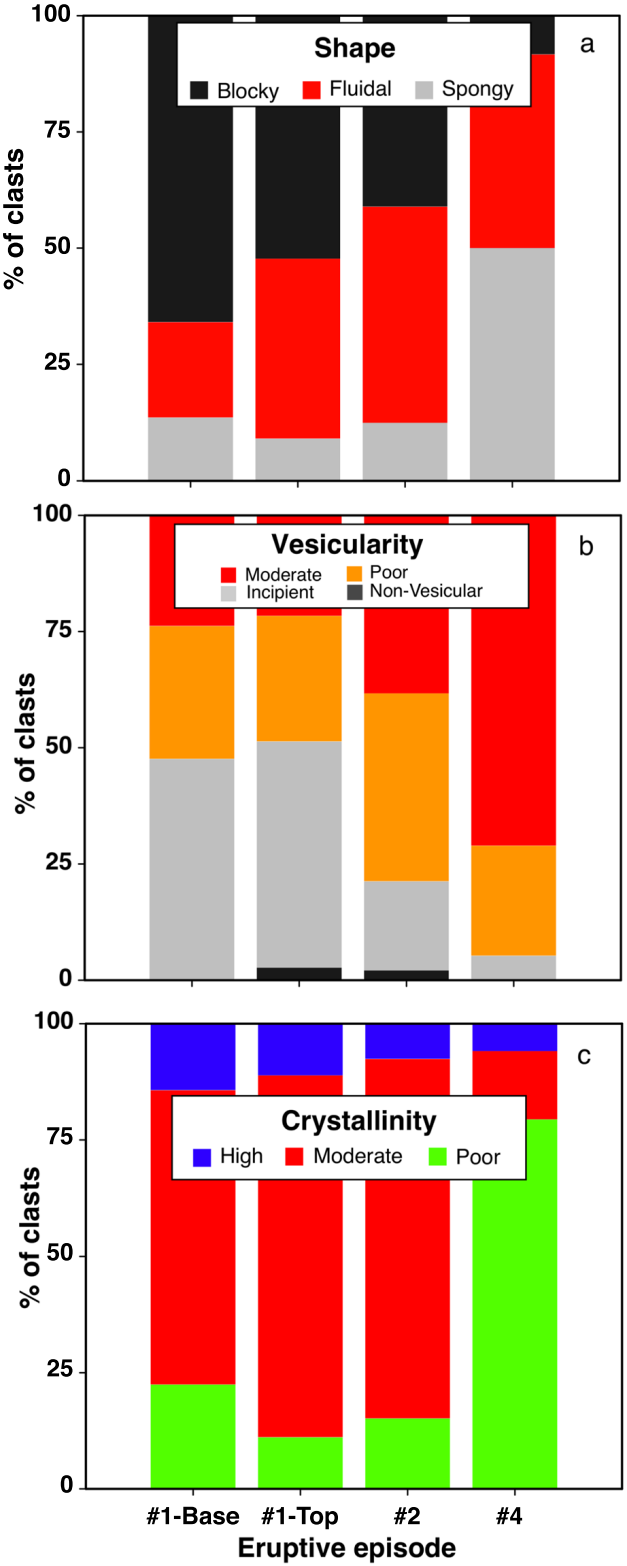
Variations in the morphological and textural parameters of the different eruptive episodes.

The relative abundances of the clast types described above vary in the deposits of the different episodes (Fig. 3a). The base of the deposit emplaced during episode #1 mainly consists of blocky clasts (66%) with minor fluidal (20%) and spongy (14%) particles, whereas in the topmost portion blocky clasts are still dominant (52%) but the abundance of fluidal clasts almost doubles (39%). In the episode #2 deposit, fluidal and blocky particles have roughly the same abundance (46% and 41%, respectively) and spongy particles are subordinate (13%). In contrast, the episode #4 deposit shows the lowest amount of blocky clasts (8%) and the highest quantity of spongy material (50%).

Vesicularity generally differs among the products of different episodes, but no significant difference is observed within the deposits of the same episode (see bottom and top of episode #1; Fig. 3b). In particular, non-vesicular clasts or clasts with incipient vesicularity diminish dramatically from episode #1 (≈50%) to episode #4 (≈5%). In contrast, the abundance of poorly to moderately vesicular particles increases from ≈50% (episode #1) to ≈95% (episode #4). Adhering dust formed by micron-sized glass shards is common in most samples.

### Groundmass texture

Products emitted from the Voragine crater during the 3–5 December 2015 explosive activity are porphyritic, as are most products of recent Etnean eruptions^12^. They contain 20–30% phenocrysts and microphenocrysts of plagioclase, clinopyroxene, olivine and Ti-magnetite set in a groundmass of varying crystallinity. Since we focused on the fine-grained portion of the deposit (1Φ), we mainly describe here textural relationships pertaining to groundmass microlites, i.e. crystals <300 µm (see discussion about size thresholds between phenocrysts and microlites of Etna products in ref. 13).

Three textural groups were identified within the ash clasts on the basis of microlite type (mainly plagioclase) and content. Poorly crystallized (PC) clasts show a glassy groundmass with rare euhedral microlites (plagioclase microlites ≤4 vol. %, Supplementary Table 2). A few microlites show a tabular shape with ratios between three axes (minimum, intermediate, maximum) of around 1–2.8–2.8 (Supplementary Table 2) estimated on the basis of CSDSlice^14^. The groundmass is on the whole homogeneous, but light-colored ribbon-like glass domains with higher atomic number are evident in some clasts (Supplementary Figure 2H).

Moderately crystallized (MC) clasts contain a glassy matrix with 5–19 vol. % plagioclase. Microlites range from tabular euhedral to hopper/swallowtail up to skeletal. Axis ratios vary from 1–7–7 to 1–9–10. Mafic crystals are absent or rare with hopper to skeletal morphologies.

Highly-crystallized (HC) clasts show a groundmass with a microlite content exceeding 20 vol. % (Supplementary Table 2). Plagioclase is mainly euhedral-tabular (axis ratios of 1-6-6 to 1-9-10, Supplementary Table 2) with minor amounts of hopper-swallowtail crystals. Olivine and clinopyroxene are generally euhedral and blocky with rare hopper morphologies.

The groundmass texture is mostly homogeneous within individual particles, but some fragments show complex textures in which HC portions are embedded within PC domains (Supplementary Figure 2I). Tiny oxide crystallites mark the interface between these distinct portions.

Episodes #1 and #2 are dominated by MC clasts with subordinate PC (16–17%) components. Episode #4 produced mainly PC clasts (79%) with minor amounts of MC fragments (<15%). HC clast contents decrease dramatically from episode #1 (15%) to episode #4 (6%). Single eruptions (episode #1) show no significant variations from top to bottom, apart from a greater abundance of PC components at the base (Fig. 3c).

### Crystal size distribution

**Figure 4:**
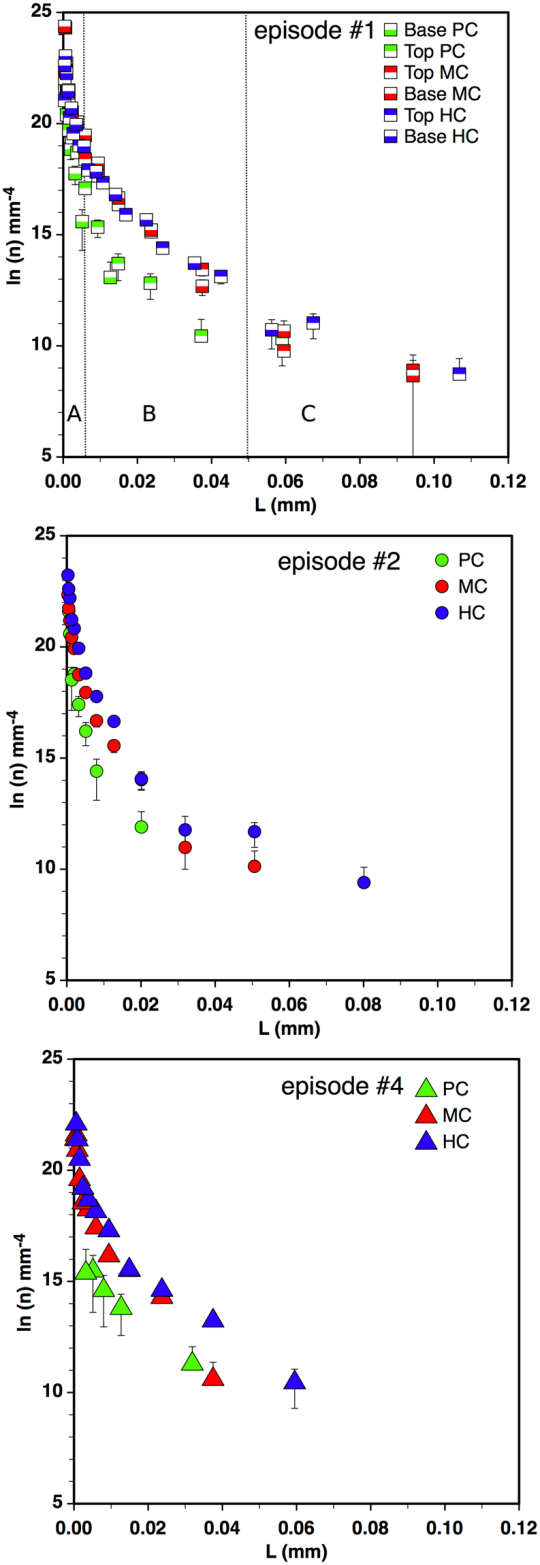
Crystal size distribution measured in clasts from different eruptive episodes (HC = highly crystalline, MC = moderately crystalline; PC = poorly crystalline). Error bars show standard deviations in crystal populations calculated according to^60^. The vertical line in the upper diagram delimits size intervals.

The crystal size distribution (CSD) is reported in Fig. 4 as classical Ln n (L) vs L plot^15^.

A common feature of all measured CSDs is the decrease in population density with increasing crystal size, describing a curve with a pronounced upward curvature. The curvature can reflect a unique lognormal distribution or a combination of simple semi-logarithmic CSDs. We can exclude on the basis of the cumulative distribution function (CDF) diagram^16^ (Supplementary Figure 3a) that measured CSDs have a pure and unique lognormal distribution, but we can consider the whole CSD as a combination of at least two segments that can be fitted with separate regression lines. The choice of the breaking points between segments can be in some way arbitrary^17^, but the use of the bi-logarithmic cumulative size plots (Supplementary Figure 3b) allowed us to identify changes in slope.

The first linear segment (A) is relative to crystal sizes <1.5–6 µm, the second (B) to those between 1.5–6 and about 50–60 µm, and the third (C) to those greater than 50–60 µm (Supplementary Table 2). Episode #4 displays only two segments, with segment B extending with a constant slope to a crystal size of about 60 µm. The largest crystal sizes were observed in MC and HC clasts from episode #1.

Segment A is present in all but one clast (PC at the base of episode #1) and shows a steep negative slope (−1400 to 3000 mm^−4^) and higher intercept (22.5–25.6) (Supplementary Table 2). In some samples (HC and MC clasts of episode #1) there is a decrease in slope or a counterslope at smaller (submicron) sizes. This feature, already observed in natural and experimental products, is real and not related to the resolution (left-hand truncation effects^18^), which is constant in all measured images.

Segment B includes a linear portion of the CSD extending from about 1.5 to 60 µm (Supplementary Table 2). Negative slopes and intercepts vary widely within these segments. For each eruptive episode, there is a progressive increase in the negative slope from HC to PC clasts, whereas intercepts do not show a regular trend. Within each textural group the CSD slopes of episode #2 clasts are generally the steepest, those of episode #1 clasts the gentlest.

Segment C is observed in most HC and MC clasts but is rare in PC particles. When present, there is a single bin-size or is affected by a large scatter in number density (Supplementary Table 2). This feature is probably related to the limited size of the area investigated (right-hand truncation effect^18^).

### Glass compositions

**Figure 5:**
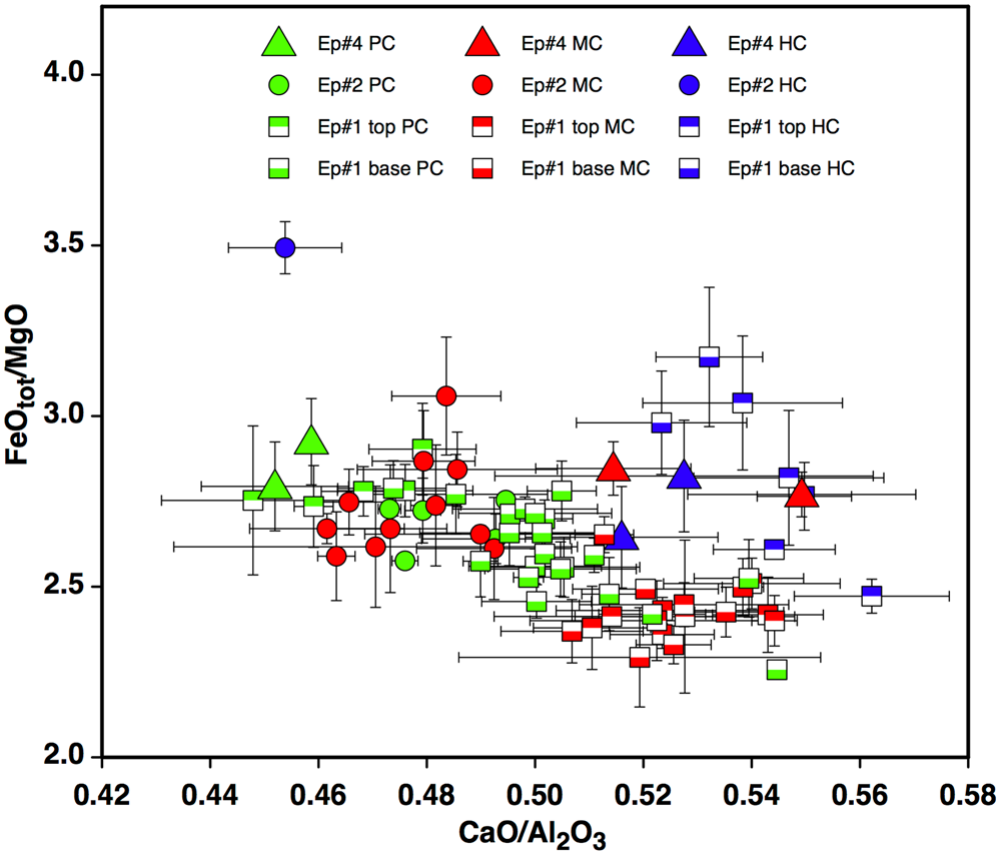
CaO/Al_2_O_3_ vs FeO_tot_/MgO diagram reporting average (~2–5 analyses per clast) groundmass compositions for clasts of differing crystallinity (HC = highly crystalline = blue symbols, MC = moderately crystalline = red symbols; PC = poorly crystalline = green symbols) from distinct eruptive episodes. Error bars show standard deviation from the average (1σ).

In the total alkali-silica (TAS) diagram glass compositions plot across the fields of trachybasalt-tephrite-basaltic trachyandesite^19^ (Table 2 and Supplementary Table 3 - Supplementary Figure 4) and partially overlap those of recent summit products from Mt. Etna. In the CaO/Al_2_O_3_ vs FeO_tot_/MgO diagram (Fig. 5), which has been traditionally used in interpreting compositional variations within Etnean magma^20–23^, analysed clasts show significant variability that exceeds the analytical error (Supplementary Table 3). Compositional variations are akin to those observed in ash erupted during earlier activity at the Voragine crater^7^. On the whole, clasts show a general negative correlation between the two ratios related to the precipitation of mafic phases dominated by clinopyroxene^20, 21^. Less differentiated parental melts plot in this diagram on the lower right angle (lowest FeO_tot_/MgO and highest CaO/Al_2_O_3_), while liquids derived by precipitation of pyroxene and plagioclase plot on the higher left angle (highest FeO_tot_/MgO and lowest CaO/Al_2_O_3_).

**Table 2:** Average composition of groundmass glass for the different textural types. HC = highly crystalline, MC = moderately crystalline; PC = poorly crystalline. # ans: number of analyses averaged. Standard deviation (1σ) from the mean is reported in brackets.

Eruption	Type	# ans	SiO_2_	TiO_2_	Al_2_O_3_	FeO_tot_	MnO	MgO	CaO	Na_2_O	K_2_O	P_2_O_5_
Episode #1 base	HC	8	49.66 (0.47)	2.21 (0.15)	15.66 (0.26)	11.23 (0.35)	0.25 (0.10)	3.66 (0.23)	8.27 (0.20)	4.36 (0.22)	3.13 (0.16)	1.05 (0.44)
Episode #1 base	MC	22	49.33 (0.41)	1.98 (0.12)	16.95 (0.33)	10.48 (0.42)	0.24 (0.16)	4.31 (0.19)	8.86 (0.23)	4.12 (0.17)	2.65 (0.14)	0.66 (0.19)
Episode #1 base	PC	76	50.00 (0.45)	1.94 (0.12)	17.07 (0.26)	10.26 (0.24)	0.21 (0.14)	3.91 (0.20)	8.52 (0.31)	4.22 (0.21)	2.71 (0.20)	0.81 (0.30)
Episode #1 top	HC	15	48.55 (0.61)	2.14 (0.17)	15.89 (0.34)	11.21 (0.48)	0.19 (0.08)	4.11 (0.32)	8.71 (0.27)	4.09 (0.17)	2.78 (0.22)	0.55 (0.08)
Episode #1 top	MC	36	48.93 (0.50)	1.94 (0.10)	16.67 (0.38)	10.35 (0.41)	0.22 (0.08)	4.29 (0.16)	8.78 (0.30)	4.03 (0.20)	2.53 (0.16)	0.52 (0.13)
Episode #1 top	PC	20	50.22 (0.27)	2.02 (0.10)	17.20 (0.27)	10.07 (0.32)	0.22 (0.10)	3.62 (0.14)	8.03 (0.19)	4.24 (0.16)	3.10 (0.11)	0.62 (0.08)
Episode #2	HC	13	49.87 (0.38)	1.97 (0.12)	16.91 (0.21)	10.22 (0.23)	0.19 (0.16)	3.82 (0.13)	8.14 (0.22)	4.11 (0.18)	2.92 (0.19)	0.92 (0.35)
Episode #2	MC	38	49.98 (0.53)	1.97 (0.13)	16.91 (0.36)	10.17 (0.48)	0.19 (0.11)	3.73 (0.14)	8.06 (0.24)	4.16 (0.20)	3.03 (0.17)	0.97 (0.38)
Episode #2	PC	3	49.93 (0.49)	2.00 (0.25)	16.40 (0.77)	10.51 (0.23)	0.29 (0.14)	3.01 (0.03)	7.44 (0.23)	3.89 (0.09)	3.41 (0.34)	0.43 (0.02)
Episode #4	HC	7	49.83 (0.77)	2.17 (0.08)	16.19 (0.46)	10.87 (0.77)	0.19 (0.10)	3.97 (0.33)	8.45 (0.35)	3.88 (0.40)	3.03 (0.25)	0.42 (0.08)
Episode #4	MC	5	47.82 (0.26)	1.91 (0.19)	15.76 (0.32)	10.84 (0.39)	0.15 (0.10)	3.88 (0.21)	8.43 (0.29)	4.04 (0.24)	2.61 (0.14)	0.58 (0.05)
Episode #4	PC	7	50.49 (0.46)	2.02 (0.08)	17.04 (0.12)	10.14 (0.25)	0.22 (0.11)	3.56 (0.10)	7.75 (0.16)	3.95 (0.17)	3.05 (0.11)	0.54 (0.07)

Compositions of analysed glasses distribute along two almost parallel trends that slightly converge at low FeO_tot_/MgO and high CaO/Al_2_O_3_. These distinct trends are also associated with textural groups identified in the groundmass. The first trend is characterised by high FeO_tot_/MgO and CaO/Al_2_O_3_, mainly ranging from 0.52 to 0.56 and corresponds to the composition of HC clasts, including those from episode #2 with the highest FeO_tot_/MgO and lowest CaO/Al_2_O_3_ values.

The second trend is associated with glasses from PC and MC fragments and shows a wider range of CaO/Al_2_O_3_ values (0.44–0.54) and FeO_tot_/MgO ratios <3.

**Figure 6:**
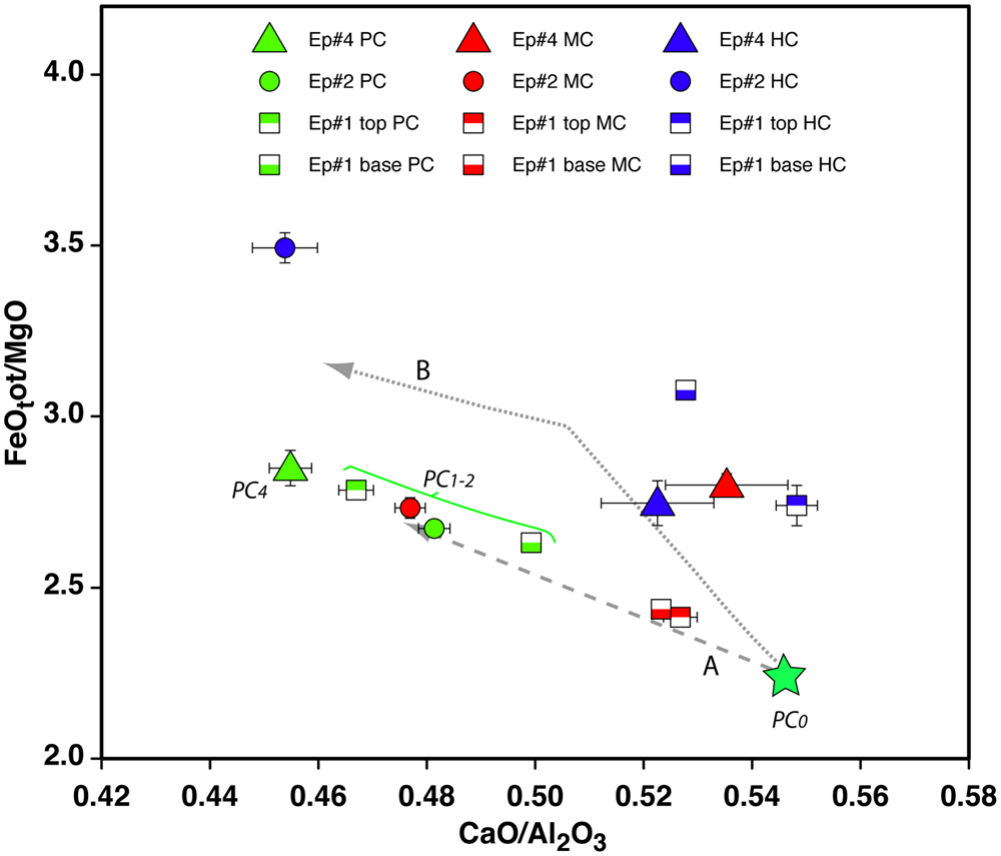
CaO/Al_2_O_3_ vs FeO_tot_/MgO diagram reporting average groundmass composition for clasts of the same type with differing crystallinity (blue = highly crystalline, red = moderately crystalline; green = poorly crystalline) from distinct eruptive episodes. Error bars show standard error of the mean. The star represents the most primitive glass composition measured in a poorly crystalline clast from the base of episode #1. This is the starting composition used for alphaMELTS modelling^24, 25^. Line A represents the liquid line of descent during crystallization of plagioclase and clinopyroxene at 0.1 < P < 25 MPa for a cooling of 30 °C under *f*O_2_ = NNO and ${f}_{{{\rm{H}}}_{{\rm{2}}}{\rm{O}}}$ = 0–1. Line B shows the liquid line of descent related to the crystallization of olivine, clinopyroxene and plagioclase at P = 0.1 MPa and T 1140–1110 °C and *f*O_2_ = QFM and ${f}_{{{\rm{H}}}_{{\rm{2}}}{\rm{O}}}$ ≈0. PC_#_ define melt compositions that refill different episodes.

Figure 6, where data on glass in clasts from distinct textural groups and distinct episodes are further averaged, reveals some additional significant differences. In particular, in episode #1: i) the average composition of glass in PC clasts is always more differentiated than that of glass in MC clasts; ii) early-erupted PC clasts are significantly less differentiated than those produced at the end of the episode, whereas MC clasts shows identical average glass compositions throughout the episode; iii) the most primitive glass composition was measured in an early-erupted PC clast.

The average composition of glass in PC and MC clasts from episode #2 plots between early and late-erupted PC clasts of episode #1. Moreover, clasts from episode #2 show the most evolved HC glass composition.

Within episode#4 the average glass compositions of PC clasts are markedly more evolved than the more crystalline counterparts (HC and MC). Glass composition measured in HC and MC clasts of episode#4 plots close to those belonging to HC clasts of episode#1.

Note that, considering the less crystallized clasts (PC) only, erupted liquids tend to become more evolved as the eruptive events progress. Thereafter on the basis of differentiation degree we will define (Fig. 6) PC_0_ as the less differentiated melt, PC_1–2_ as the intermediate melts, and PC_4_ as the most evolved liquid.

### Magmatic processes

AlphaMELTS code^24, 25^ was employed to simulate melt-mineral equilibria and liquid evolution in a P-T grid (P: 0.1–25 MPa; T: 1200–1090 °C, step intervals of 5 MPa and 5 °C) with variable *f*O_2_ (QFM-NNO) and ${f}_{{{\rm{H}}}_{{\rm{2}}}{\rm{O}}}$ (0–1) representing expected physical conditions within the shallow magmatic system of Mt. Etna and eruptive temperature measured in recent eruptions^7^. The composition of the starting melt was set to that of the less evolved glass (PC_0_ with the highest FeO/MgO and lowest CaO/Al_2_O_3_) (Fig. 6). The liquidus temperature (T_liquidus_) and particularly the appearance of plagioclase are strongly controlled by water content. At ${f}_{{{\rm{H}}}_{{\rm{2}}}{\rm{O}}}$ = 1 pyroxene is the first phase to appear (T = 1100–1105 °C) at P > 5 MPa. Plagioclase is absent at P = 20 MPa, is the second phase (T = 1100 °C) at P = 15 MPa and is the first phase (1130 °C and 1160 °C) at P = 5 MPa and 0.1 MPa respectively.

At ${f}_{{{\rm{H}}}_{{\rm{2}}}{\rm{O}}}$ ≈0 plagioclase is always a liquidus phase with a quite constant T_liquidus_ (1135°at P ≥ 5 MPa) that attains its highest value of 1160 °C at atmospheric pressure.

Taking into account the phase relationships described above, the expected range of possible effective undercooling (Δ*T*_*e*_ = T_liquidus_-T_magma_) within the shallow plumbing system remains narrow (≈35–70 °C) but varies during ascent. For example, assuming dry melts and an almost isothermal conduit (with T_magma_≈T_eruptive_≈1100–1090 °C) extending down to P=25 MPa, the Δ*T*_*e *_remains constant at around 35 °C throughout the conduit and reaches the maximum of 60 °C at the surface. In contrast, during ascent, degassing water-saturated melts undergo a progressive increase in Δ*T*_*e*_ that become pronounced (≈40–70 °C) close to the surface.

Compositional changes in groundmass glass were compared with the melt compositions obtained through alphaMELTS (Fig. 6) simulations within the investigated P-T field. PC and MC glass compositions mainly plot along a liquid evolution line produced by crystallization of plagioclase and clinopyroxene at 0.1 < P < 25 MPa for <30 °C cooling under *f*O_2_ = NNO and ${f}_{{{\rm{H}}}_{{\rm{2}}}{\rm{O}}}$ = 0–1 (Line A in Fig. 6). In contrast, the average composition of HC clasts plots along a liquid line of descent related to the crystallization of olivine, clinopyroxene and plagioclase at P = 0.1 MPa and T 1140–1110 °C and *f*O_2_ = QFM and ${f}_{{{\rm{H}}}_{{\rm{2}}}{\rm{O}}}$ ≈0. Consequently, the observed trends are on the whole related to melt evolution and microlite crystallization as a consequence of slight cooling (<30 °C) and degassing at shallow depth (P < 25 MPa) during the last stages of pre-eruptive stationing of the magma. In particular, some HC clasts are produced by microlite crystallization from an almost degassed melt very close to the surface.

### Rheology

**Figure 7:**
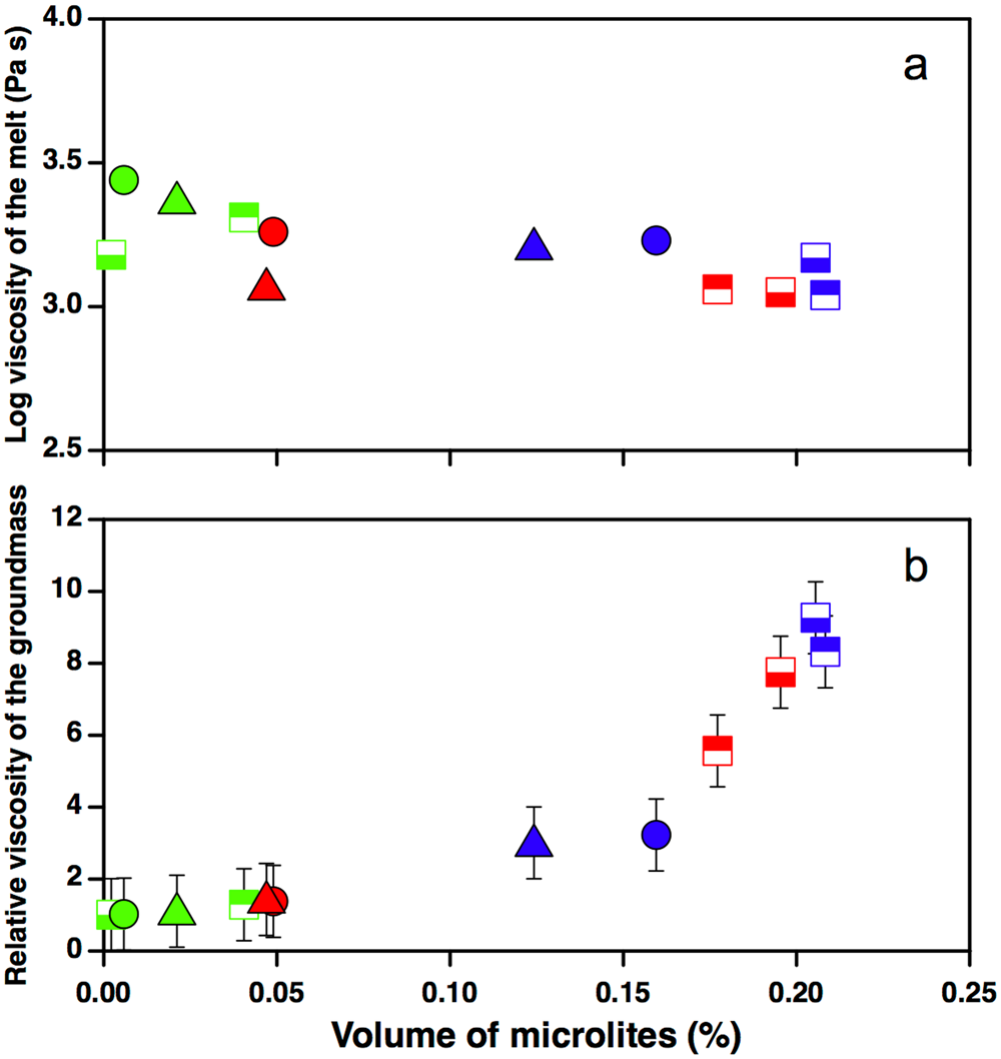
(**a**) Log_10_ of the Newtonian viscosity determined only for the melt phase at 1100 °C using the GRD model^61^ within clasts with a variable microlite content; (**b**) relative viscosity of the groundmass (melt + crystals) as a function of crystal fraction calculated on the basis of eq. 23 of the^29^ model, using an average crystal aspect ratio of 2.5 to 9.5, shear rates (4.8 10^−3^–7.4 10^−4^ s^−1^) were calculated from MDR estimated by^8^, assuming 1000 m-long conduit with a radius of 20 m.

Magma rheology is controlled by changes in chemical composition and by variations in crystal and vesicle content and shape^26–28^. The Newtonian viscosity of the melt, calculated at a relevant temperature (1100 °C) following the GRD model^28^ (Fig. 7), decreases slightly with increasing crystallinity. Although surprising, this agrees with the composition of HC and MC clasts, in particular those of episode #1, which show lower SiO_2_ and Al_2_O_3_ concentrations with respect to PC clasts. The above trend is totally reversed if the relative viscosity calculated according to^29^ is reported as a function of crystal fraction. The high aspect ratio (~6–9.5; Table 2) and volume of microlites observed in HC and MC clasts of episode #1 produces a sixfold to tenfold increase in apparent viscosity (Fig. 7).

HC clasts of episodes #2 and #4, with a slightly lower crystal fraction (0.12–0.16), indicate a significantly lower increase in relative viscosity (~threefold). The low microlite content in PC clasts from all episodes and MC clasts from episode #2 and #4 is such that the relative viscosity reaches values of up to 1.5.

This general pattern does not change significantly if we consider the effect of the bubbles on the relative viscosity of the magma. According to the review of Mader *et al*.^27^ changes of relative viscosity due to variable vesicle content or considering monodisperse or polydisperse size distribution, don’t exceeds a factor 2 and are thus negligible with respect to sixfold to tenfold increase in apparent viscosity due to the microlite content and shape.

## Discussion

Textural and compositional data highlight the large variability of products both within single episodes and among different ones. Textural and compositional heterogeneities, widely documented in the products of explosive basaltic eruptions from several volcanoes, have been ascribed to pre-eruptive storage and ascent and/or flow conditions during the eruption^4, 6, 30–35^. Since this work focuses on the last stage of magma ascent, we did not investigate the record of early, deep magmatic processes contained in phenocrysts and their melt inclusions.

Major differences among the studied samples pertain to composition, crystallinity, vesicularity and external shape of clasts. These properties are closely related. Groundmass crystallization has a strong control on glass composition and can dramatically affect rheology (viscosity, yield strength), which in turn controls fragmentation style and the shape of clasts. This process is particularly relevant in low viscosity, high temperature basaltic melts, where crystallization of microlites can promote notable rheological changes (more than one order of magnitude)^36^. Textural observation of tephra from recent explosive basaltic eruptions of varying style (from Strombolian to Plinian) suggest that increased viscosity can induce brittle fragmentation and may control the transition from mild to more violent phases^32, 37^. Physical modelling also confirms (see Fig 4.10 in ref. 38) that the transition to the brittle fragmentation occurs at increasing viscosity and mass discharge rates. Bubble content, melt-bubble coupling and resulting magma permeability also control the strength of the magma and fragmentation mechanisms, modulating the transition between eruptive styles^39^.

Below we use information derived from melt composition, crystal size distribution, and rheological properties to reconstruct how and when these processes developed in time, both before and during the eruptive episodes.

To understand how the rheological and physical properties of the magma within the conduit vary during a single eruption or throughout the eruptive sequence, we also assume that the abundance of different textural components reflects distinct volumes of magma within the magmatic plumbing system.

Glass represents the melt phase of the magma stored within the conduit; its composition reflects late stage crystallization processes (groundmass and phenocryst rims) and may also be controlled by mixing between melts produced by the above processes.

Comparison between the simulated liquid line of descent and natural compositions reported in Fig. 6 reveals that the glass composition of HC clasts (Trend B) is related to groundmass crystallization from a totally degassed melt at depths/pressures close to the surface. These clasts likely represent magma in the upper part of the column or sluggish magma batches located along the conduit walls. They may also represent dense blobs of an early degassed, solidified magma sinking within the conduit.

MC and PC glasses of episodes #1 and #2, in contrast, reflect the melt evolution and crystallization of a degassing magma at slightly higher pressures (<25 MPa). In Fig. 6, the crystallization of plagioclase and clinopyroxene produces residual glasses with progressively higher FeO/MgO and lower CaO/Al_2_O_3_ (trend A). This simple process can account for the similarities in MC and PC glasses of episode #2 but not for the differences between PC and MC glasses of episode #1. MC glasses erupted at the beginning and end of episode #1 show higher CaO/Al_2_O_3_ and lower FeO/MgO than PC clasts erupted at the same time. This implies the existence of a parental melt with an even higher CaO/Al_2_O_3_ and lower FeO/MgO, which could be represented by the composition of some rare clasts from episode #1 (PC_0_-green star in Fig. 6). The same parental melt has produced HC and MC clasts erupted during episode #4.

The low microlite content of PC clasts erupted during the subsequent eruptive episodes and their relative position in Figs 5 and 6 indicates that their compositions (PC_1–2_ and PC_4_) are not related to crystallization of the groundmass but reflects progressive evolution, through crystal fractionation (phenocrysts), of a magma batch presumably stationing in a different, deeper magmatic environment. This magma differentiated under conditions of slight undercooling which favours growth rather than nucleation. The progressive migration of magma between magmatic environments on Etna has been described in detail by^40^ even at the scale of a single eruption. Conditions pertaining to conduits and those related to a deeper reservoir may represent, respectively, the ME_2_ and ME_1_ magmatic environments defined by^41^.

CSD is an important tool for deciphering the crystallization history of magmas, including the time scale of processes^18^. A concave-upward shape of the resulting CSD pattern (Fig. 4) is quite common in the products of Etna and other volcanoes, as well as in experimental charges. This feature has been interpreted as the result of different processes, including textural coarsening, crystal agglomeration or accumulation, or mixing between different crystal populations^13, 42–46^. We here consider only microlite sizes <150 µm, which are supposed to nucleate and grow during the last stages of pre-eruptive ascent and during the eruption. We can thus exclude that processes such as crystal agglomeration or accumulation have significantly affected measured CSDs. Similarly, we exclude textural coarsening for larger crystals since this process is favoured by annealing of the crystals over long periods^47^. More frequently, a curved CSD has been interpreted as a combination of populations generated during a complex magmatic history in which phases of crystallization takes place under distinct and variable conditions (mixing^48^). In this view, the distinct segments account for abrupt changes in physical or chemical conditions that control kinetic parameters (e.g., nucleation and growth rate). The whole CSD can therefore be read as sequence of magmatic and volcanic processes occurring before and during the eruption.

Consequently, the segment of the CSDs (A in Fig. 4) with the smallest crystal sizes, which is similar for most samples, is likely related to the strong thermal perturbation occurring during post-fragmentation quenching. Considering the homogeneous, limited size/mass of the analysed clasts, we suppose that quenching operated in the same way on all clast types in all the eruptive episodes thus producing a comparable CSD. These microlite sizes are those expected for a reasonable quench rate, from T_eruptive_ to T_glass transition_, for fine fragments (20° K s^−1^) and a fast growth rate (G) (1.85 10^−9^ m s^−1^)^43, 49^.

In contrast, the B segments show larger variability in slope and intercepts, reflecting different conditions and related changes in crystallization parameters during the pre- and syn-eruptive stages.

Segment C, when present, is sparsely populated due to the small size of the area analysed and represents an early stage of crystallization possibly within a deeper reservoir. However, the paucity of data available does not allow for a fully reliable interpretation.

As a consequence, the information on pre- and syn-eruptive magmatic processes is derived mainly from variability observed within segment B. In particular, the most critical parameter that can be derived from crystal size is the time of residence. Since crystallization is related to both cooling and degassing during ascent, if all the magma batches evolve in the same temperature interval or follow an ascent path of the same length, different residence times will correspond to varied cooling or ascent rates.

The slope and intercept of CSDs depend on the growth rate (G), nucleation rate (J) and their variation in time^18, 50^. Assuming that the growth rate remains constant within the same eruptive episode for slight undercooling^18^, the differences in slope can be attributed mainly to variations in the residence time of magma batches and the resulting development of different kinds of clasts.

Therefore, in the first two episodes, the fact that the slopes of PC clasts are two-three times steeper than those of the corresponding MC and HC clasts would suggest that glassy PC clasts experienced faster cooling or shorter residence times with respect to MC and HC clasts.

Figure 4 shows that there are also significant differences among clasts of the same type from different eruptive episodes. In particular, all clast types from episode #2 show higher negative slopes than clasts from episodes #1 and #4. We ascribe this feature to the shorter residence time in the conduit of magma batches that fed episode #2.

Assuming that the size interval observed in segment B is representative of crystallization within the conduit, it is possible to infer the residence time of magma in this part of the plumbing system before fragmentation and quenching. To this end, we need to assume reasonable values of the growth rate. Values of G reported in literature for Etna magmas vary of several order of magnitude (G = 10^−7^–10^−11^ m s^−1^ ^43, 51, 52^). This variability produce a comparable wide range of residence times that spans from 300 seconds to about 1 month, considering the whole extent of B segment (≈60 microns). This incertitude can be reduced taking into account that HC and MC clasts of episode #2 result from the crystallisation of a melt that arrived in the conduit at the end of episode #1 (≈30 hours before). Thus, assuming that the maximum length (L_max_) in segment B of HC and MC clasts in episode #2 has been attained in about 30 hours, a growth rate of about G = 1.5 10^−10^ m s^−1^ results.

We are considering here the maximum length (L_max_) instead of average length (L_med_ = 1/slope) of segment B, since L_max_ is the length of a crystal that has continued to grow for the entire characteristic crystallization period for that segment^44, 53^.

Under these assumptions, using L_max_ and the above calculated G, for episodes #1 and #4, we infer that microlites in HC and MC clasts developed at least within 34 to 55 hours before the eruption, whereas those in PC clasts formed between 8 and 11 hours.

Thermodynamic modelling reveals that compositional differences are compatible with crystallization by cooling or degassing at P < 25 MPa, corresponding to a depth of about 1 km below the volcano summit according to the rock density profile proposed by^54^. This depth is consistent with the conduit extension and with the shallower portion of the Mt. Etna plumbing system as imaged by seismic data (in particular volcanic tremor and LP and VLP signals^55^). Compositional variations in glass also suggest the occurrence of deeper magma storage in which phenocrysts form and grow, producing a variably differentiated residual melt. In the shallower portion of the plumbing system, the crystallization of microlites due to cooling or degassing also leads to important changes in the rheological properties of the magma. Collected data indicate that these rheological changes can occur within tens of hours, thereby affecting syn-eruptive processes, including fragmentation mechanisms and determining the shape and surface morphology of pyroclasts. This is consistent with variations in the morphology, vesicularity and crystallinity of particles observed in the products of the different eruptive episodes.

**Figure 8:**
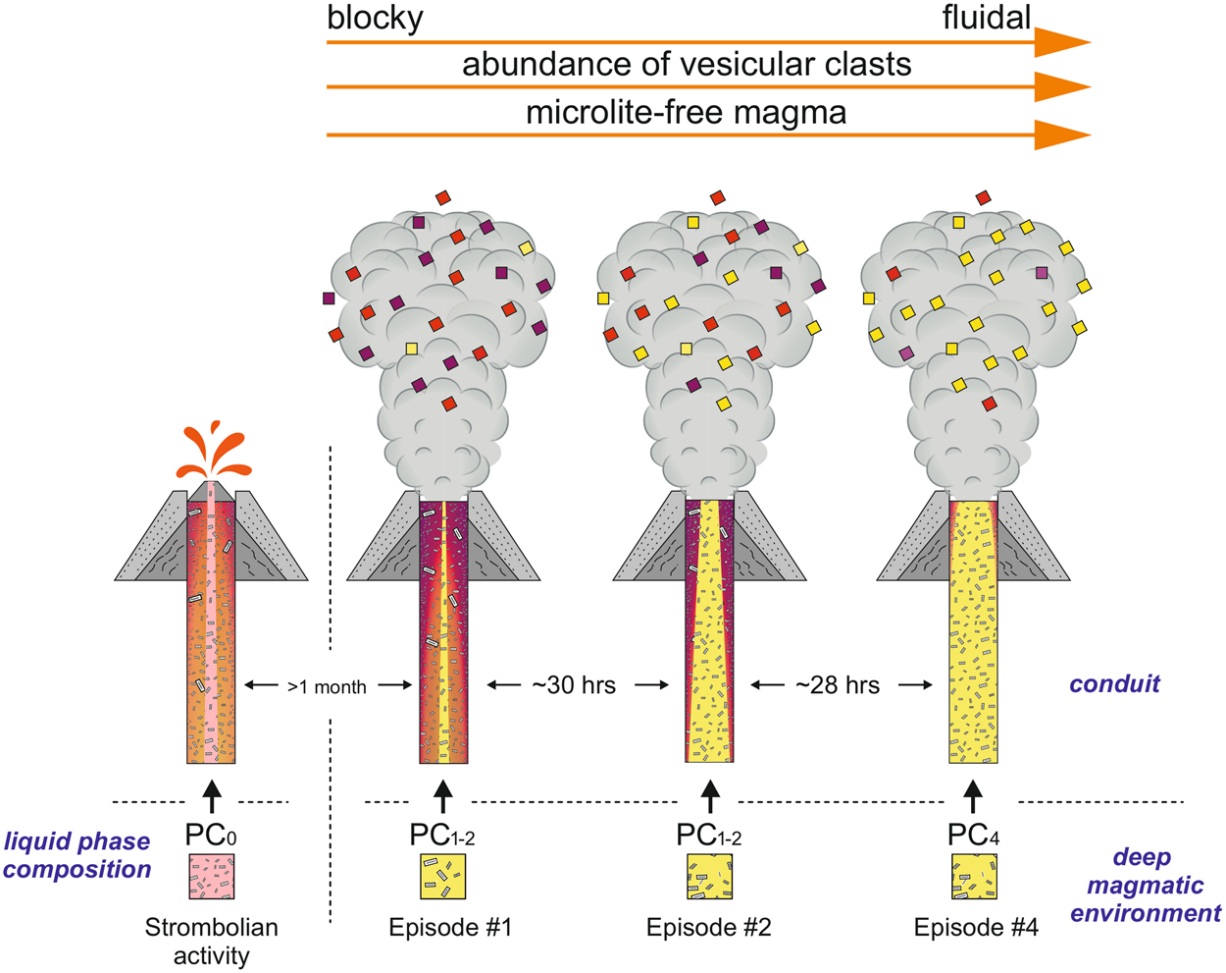
Interpretative model of magmatic and volcanic processes responsible for the four eruptive episodes in December 2015.

In the following we propose a conceptual model that integrates magma dynamics, rheological changes and eruptive processes occurring within the shallow portion of the plumbing system (Fig. 8).

In the second half of October 2015 an early ascent of magma progressively reopened the conduit of the Voragine feeding the weak intra-crater Strombolian activity. The arrival of fresh magma in the conduit possibly increased just before the episode #1. At that moment, the magma column consisted of a dominant volume with variable microlite content (Fig. 8). CSD and compositional data show that microlites reflect shallow crystallization and degassing from a magma that has arrived in the Voragine conduit at least 52 hours before the onset of episode #1. The liquid phase of this magma has the most primitive composition observed in the products of the whole eruptive period and is preserved only in rare clasts (PC_0_ star symbol in Fig. 6).

In this period of time the magma experienced effective volatile exsolution, bubble separation, and microlite crystallization. Less than 12 hours before the eruption a volumetrically subordinate microlite-free magma batch (<25% of the volume) representing a more evolved melt was emplaced in the conduit and possibly mixed with the resident magma, producing the compositional zoning observed in the residual melts of episode #1 (late products are more evolved than the early ones). This new input of magma determined the change of the eruptive behaviour from mild Strombolian to violent explosive and triggered the brittle fragmentation of resident magma (50–60% of particles have a blocky shape). The brittle fragmentation was possibly favoured by magma viscosity that had significantly increased (six- to tenfold with respect to melt viscosity) due to the crystallization of microlites.

In the episode #2, a dominant volume with variable microlite content was still stationing in the conduit, but **i**n this case, microlites are related to the crystallization (by cooling or degassing) of a melt with an evolved composition similar to that of the melt that entered the conduit at the end of episode #1 (PC_1–2_ Figs 6 and 8). This magma has produced microlites for about 30 hours before the beginning of episode #2. A subordinate volume of a microlite-free magma batch (<15% of the total volume) having a composition that partially overlaps with that of magma erupted at the end of episode #1 was also emitted. The emplacement of this magma occurred less than 8 hours before the eruption. The vesicle content of the magma feeding episode#2 was higher than in episode #1 since more than 50% of the clasts are poorly or moderately vesicular. The number of blocky fragments decreases to less than the 40% of the volume, and fluidal shapes are more abundant indicating a more pronounced “inertial fragmentation”. This is in agreement with the lower relative viscosity calculated for magma forming the HC and MC clasts of this eruptive episode.

During episode #4, the volume of highly viscous microlite-rich magma is limited (<25% of the total volume) and the magma column was dominantly filled (more than 75%) by a microlite-free melt. The composition of this melt, emplaced around 12 hours before the eruption, is the most evolved one of all products erupted in this period (PC_4_ in Fig. 6). The portion consisting of crystallized magma (MC and HC) was emplaced or started to crystallize more than 2 days before the eruption at very shallow depths from a liquid with an intermediate composition between PC_0_ and PC_1–2_. These clasts might represent subordinate magma pockets not erupted during previous episodes. More than 90% of the clasts produced during this episode are poorly to moderately vesicular and have a fluidal or spongy shape. This corresponds to a magma column with zones where fragmentation of the low-viscosity melt was inertially driven by a sustained gas outflow (fluidal particles) and zones where a gas-melt coupling is more efficient favoring the formation of spongy clasts.

In conclusion, the combined textural and compositional characterization of ash reveals that the magmatic column feeding explosive episodes at Mt. Etna is strongly heterogeneous. In the studied paroxysms there is always the contemporaneous occurrence within the conduit of a high viscosity portion with a variable content of microlite and a less viscous volume of microlite-free, gas-rich magma.

During each single episode these heterogeneities can develop in few tens of hours. The time scale for the total refilling of the system and the renewal of magma is in the same order of magnitude (e.g. 30 hours between episode 1 and 2).

The composition of these ascending magma batches changes in time and becomes progressively more evolved, as deeper crystallizing stores of magma are tapped. This behaviour, though not unusual in Mt. Etna’s shallow plumbing system, as outlined by Khal *et al*. in their studies^40, 41^ of olivine zoning patterns and eruptions, is markedly different from those proposed by^7, 56, 57^ for summit explosive activity on the basis of bulk chemistry and by^58^ on the basis of ash glass composition.

Our analysis also confirms that the amount and shape of microlites, together with melt composition, have a strong control on rheological properties.

On this basis, we suggest that the transition between weak intracrater Strombolian activity and paroxysmal phases with km-high sustained columns, could be related to relative proportions within the conduit between high (microlite-rich) and low (microlite poor-gas rich) viscosity portions.

As shown in previous explosive eruptions of Mt. Etna^31, 32^, the ratio between these two components control fragmentation style and plume height. The prevalence of a crystalline volume favours brittle fragmentation and higher column heights. Nevertheless, the gas-rich microlite-free magma batches play an essential role because propel the explosive eruptions.

This work confirms that ash studies represent a powerful tool for unravelling the details of eruption dynamics. Combined textural and compositional investigations of ash, whose time of the eruption is well known, are crucial in this respect. In addition, our results indicate that compositional information from a single ash component can be misleading and that all components should be analysed in order to gain detailed information on magmatic columns and the development of eruptive processes. We also wish to stress that bulk chemistry, traditionally employed for petrological monitoring, may not be very informative in the analysis of such phenomena.

## Methods

Deposits were sampled a few days after the eruptive crisis. Specimens were collected along the dispersal axes at 6 to 14 km from the vent. Sampling was carried out exclusively on horizontal man-made surfaces (e.g.: roofs, terraces, walls) to prevent any contamination or mismatch with tephra from previous eruptive episodes. For the same reason, we did not sample deposits from episode #3 due to its distribution on an area intermediate to that of episodes #1 and #2. A total of 23 samples were collected from 11 sampling sites (Fig. 1, Supp. Table 1). In sites 1 and 2, the thickness of the tephra bed (4–5 cm) allowed detailed vertical sampling with a sampling interval of about 1 cm. Grain size analysis was performed on 11 samples by dry sieving at 1Φ interval (Φ = −log_2_D, where D is the particle diameter in millimetres), for the −4Φ to 5Φ grain size range. Seven samples were selected to investigate clast morphology and texture and the composition of the groundmass.

In the case of samples from episodes #1 and #2, about 50 clasts from the 1Φ grain size fraction were randomly hand-picked and mounted on a glass slide. Because the sample of episode #4 has a finer grain size, we mounted only the 2Φ fraction. To test the representativeness of the episode #4 sample and to avoid possible bias derived from the different grain sizes, we also observed the 2Φ fraction of one episode #1 sample for comparison. The clasts were then analysed with a Zeiss EVO M10 Scanning Electron Microscope (SEM) at the INGV-Pisa. After ash morphology characterization, slides were impregnated with epoxy resin and prepared as standard thin sections to obtain internal textural and compositional data through BSE imaging and microanalysis.

About 250 glass analyses were completed in raster mode (squares 5–10 microns in length) using an ISIS-Oxford microanalytical system linked to the SEM. Data were collected at an acceleration voltage of 15 Kev, using a 300pA current probe with an acquisition time of 100 sec. Concentrations were obtained after ZAF correction using natural minerals or pure oxides for calibration. With few exceptions, for each clast three analyses were completed and averaged. To check the precision and accuracy of obtained data, VG-2^59^ standard glass was analysed several times (60 analyses) before and after the different analytical sessions.

Precision values, expressed as a percent relative standard deviation (RSD%) are Na_2_O = 3.4, MgO = 2; Al_2_O_3_ = 1; SiO_2_ = 0.9; CaO = 1.75; TiO_2_ = 5.2; FeO_tot_ = 1.9.

To further increase homogeneity between different analytical sessions and to allow comparison with other published data sets, raw concentrations were normalised to the VG-2 reference value of standard glass as follows:

1 $${C}_{norm}^{i}=\,{C}_{measured}^{i}\times {k}_{norm}^{i}$$

2 $${k}_{norm}^{i}=\,\frac{{C}_{standard\,reference\,value}^{i}}{{C}_{standard\,session\,average}^{i}}$$

with i= SiO_2_, TiO_2_, Al_2_O_3_ etc.

${k}_{norm}^{i}$ are always in the 0.9–1.1 range

Crystal size distribution (CSD) was determined on 12 samples representatives of all clast types and eruptive episodes. Back-scattered electron (BSE) images were collected from polished thin sections of the clasts at 1000 x magnification with a resolution of 3072 × 2304 pixels. This corresponds to an area of 0.26 × 0.19 mm for a square pixel measuring 0.08 microns per side. Images were then binarized in order to select plagioclases and manually adjusted to separate touching crystals and reduce noise. 2D Area, long and short axes of best fitting ellipses of microlites were measured through ImageJ freeware, excluding particles touching the border and those with an area of less than 10 pixels. This allowed us to detect submicron particles with confidence (minimum area 0.64 square microns).

## Electronic supplementary material

Supplementary figures

Supplementary Table 1

Supplementary Table 2

Supplementary Table 3
