# Supplementary material for: Magma dynamics within a basaltic conduit revealed by textural and compositional features of erupted ash: the December 2015 Mt. Etna paroxysms
Source: Sci Rep. 2017 Jul 6;7:4805. doi: 10.1038/s41598-017-05065-x (PMC5500471; doi:10.1038/s41598-017-05065-x)
Supplement: Supplementary file 1 — Supplementary figures [file 41598_2017_5065_MOESM1_ESM.pdf]

Magma dynamics within a basaltic conduit revealed by textural and compositional features of erupted ash: the December 2015 Mt. Etna paroxysms

Massimo Pompilio, Antonella Bertagnini, Paola Del Carlo, Alessio Di Roberto

Istituto Nazionale di Geofisica e Vulcanologia, Sezione di Pisa (Italy)

Supplementary Information

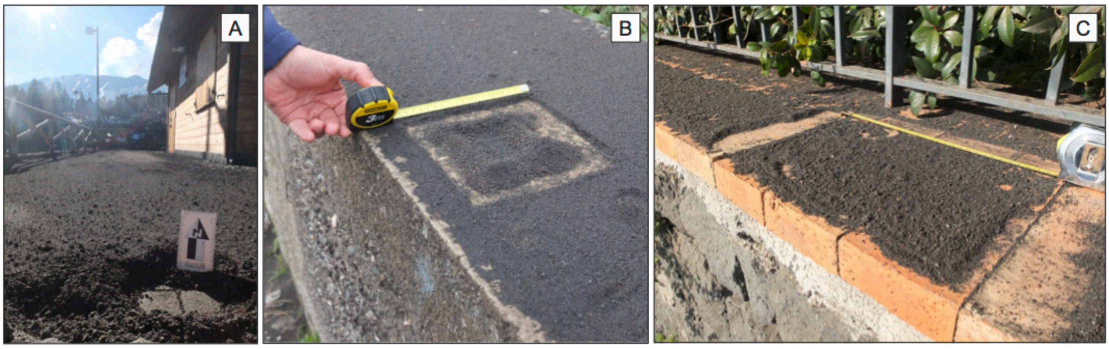

Supplementary figure 1: A: episode #1 deposit from site S1 (see Figure1), located along the dispersion axis at 6.5 km from the vent; B: episode #1 deposit from site S6 (see Figure1) located along the dispersion axis at 13 km from the vent. The square measures 20 cm per side; C: deposit of episode #2 at site S8 (see Figure1) located along the dispersion axis at about 12 km from the vent; the ruler is 30 cm long.

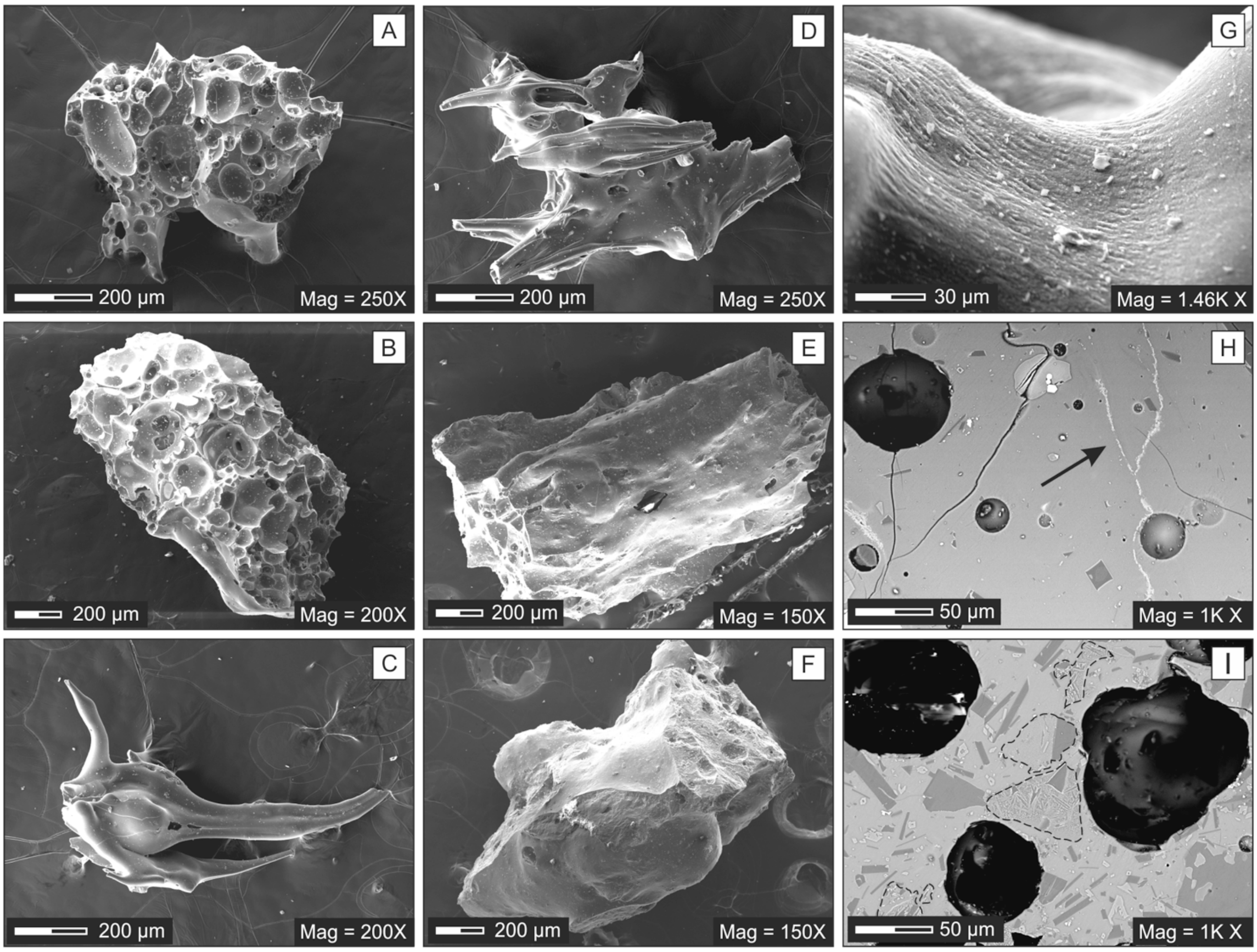

Supplementary figure 2: SEM images of the external morphology and internal textures of clasts. A and B: spongy; C and D: fluidal; E and F: blocky; G: wrinkles on fluidal clast surface; H: BSE image, with arrow pointing at light-coloured ribbon-like glass domains with higher atomic number; I: HC portions embedded within PC domains.

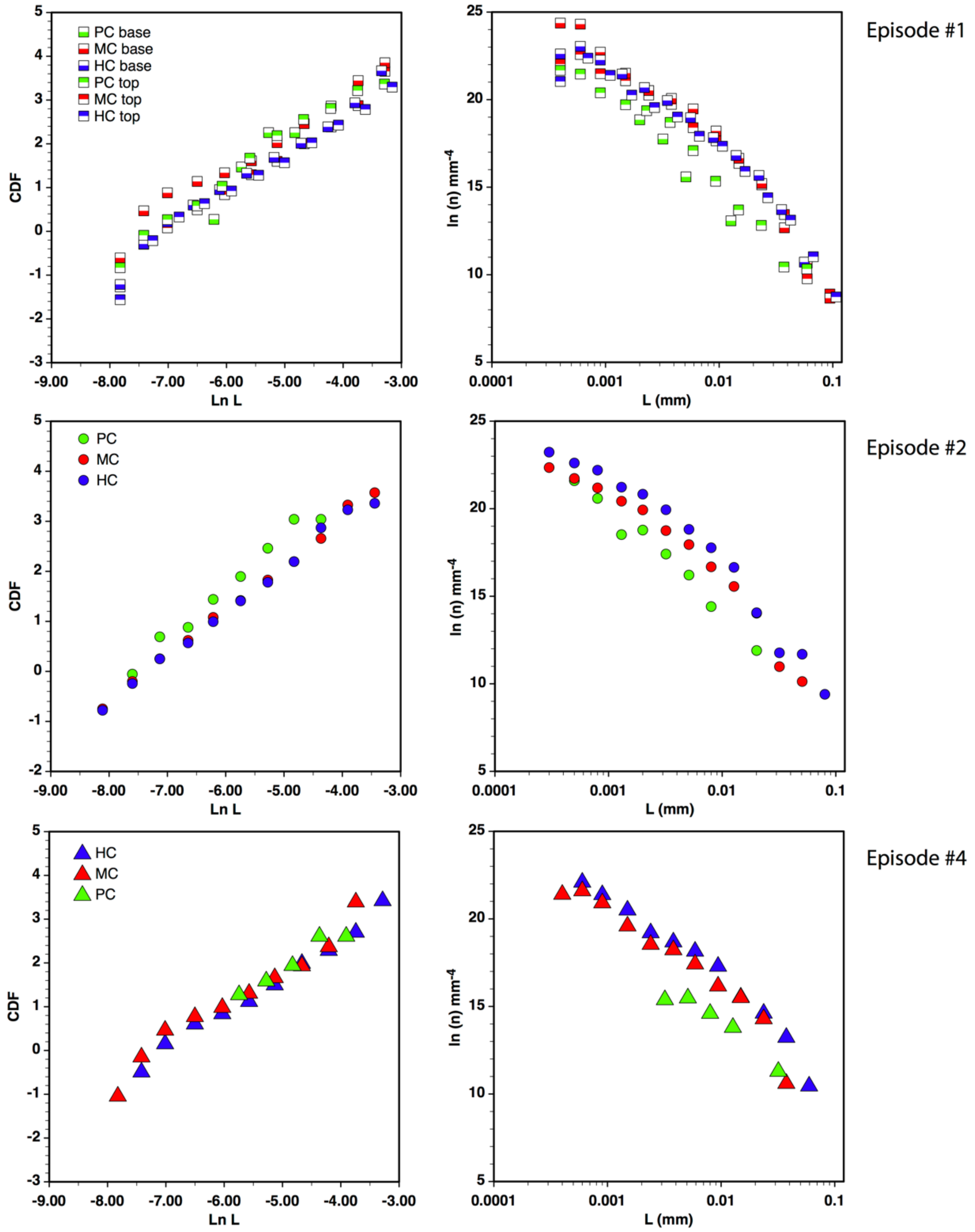

Supplementary figure 3: Crystal size distribution measured in clasts from different eruptive episodes (HC= highly crystalline, MC= moderately crystalline; PC=poorly crystalline) plotted in left column as cumulative distribution function (CDF) vs Ln L (length of crystals in mm) and in right column as bi-logarithmic cumulative size. On CDF diagram a single lognormal distribution plots as straight line symmetric across the centre of the distribution. On bi-logarithmic cumulative size diagram a single lognormal distribution plot as a flat curve at small sizes[16].

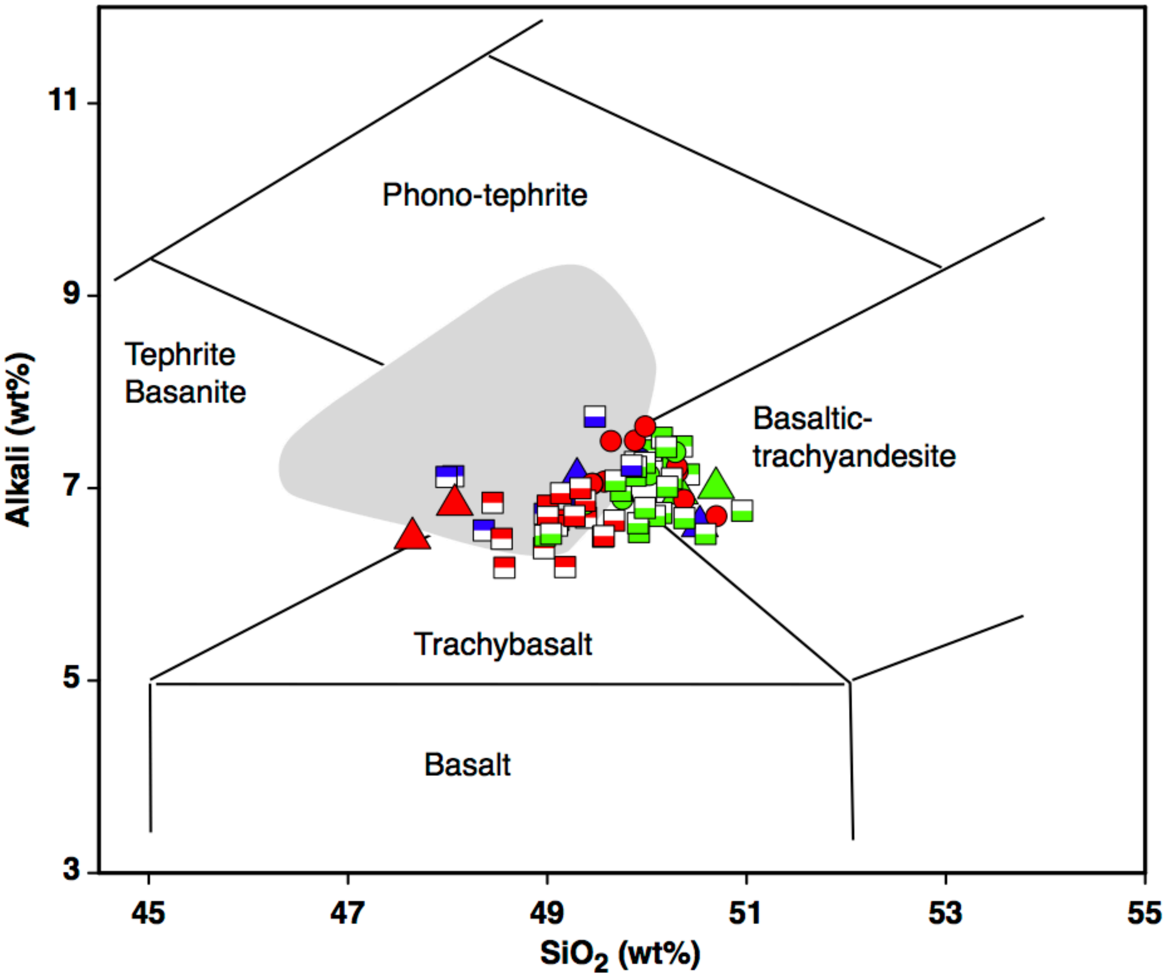

Supplementary figure 4: Total Alkali Silica diagram. Symbols as in figure 5, HC= highly crystalline= blue symbols, MC= moderately crystalline= red symbols; PC=poorly crystalline= green symbols. Grey area represents composition of glass in tephra from recent eruptions of Mt. Etna (from[4,7]).
